# Is the Central Sensitization in Chronic Nonspecific Low Back Pain Structural Phenomenon or Psychological Reaction? A Narrative Review

**DOI:** 10.3390/jcm14020577

**Published:** 2025-01-17

**Authors:** Józef Alfons Opara, Edward Saulicz, Jarosław Wojciech Szczygieł, Katarzyna Szczygieł

**Affiliations:** 1 Institute of Physiotherapy and Health Sciences, Jerzy Kukuczka Academy of Physical Education, ul. Mikolowska 72b, 40-065 Katowice, Poland; e.saulicz@awf.katowice.pl (E.S.); j.szczygiel@awf.katowice.pl (J.W.S.); 2 Department Neurology, Clinical Hospital No. 1 Named After Prof. Stanisław Szyszko, ul. 3 Maja 13/15, 41-800 Zabrze, Poland; 3 Department Neurology, Upper Silesian Medical Center named After Prof. Leszek Giec, ul. Ziołowa 45/47, 40-635 Katowice, Poland; kbszczygiel@gmail.com

**Keywords:** central sensitization, chronic nonspecific low back pain, CNLBP, allodynia, assessment

## Abstract

Lower back pain (LBP) is a common condition affecting primarily populations in developed countries, placing a significant burden on public health systems around the world. A high rate of pain recurrence increases the risk of developing a chronic syndrome and the occurrence of complex psychosocial and professional problems. Symptoms lasting longer than 12 weeks are associated with the risk of sleep problems, depression, and anxiety. In the 21st century, the development of knowledge about central sensitization (CS) to pain allowed for a better understanding of the pathomechanism of pain and the transformation of LBP into a chronic form. Patients with chronic severe pain often experience accompanying symptoms such as fatigue, depression, anxiety, sleep disorders, appetite disorders, flatulence, dyspepsia, and dizziness—these are part of the picture of CS. The article presents a narrative, chronological review of reports covering the current state of knowledge on the possibility of assessing central sensitization and its impact on low back pain. The authors also attempt to answer the question posed in the title. CS can be seen as an excessive reactivity of nociceptive neurons in the central nervous system to normal or subthreshold afferent chronic stimuli in people with certain mental predispositions.

## 1. Introduction

According to a commonly accepted definition, lower back pain (LBP) is a symptom, not a disease, and is usually located between the lower edges of the ribs and the gluteal folds [1]. LBP is often accompanied by pain in one or both lower limbs; some people may present with neurological deficits in these limbs that justify the diagnosis of radiculopathy. Members of the Lancet Low Back Pain Series Working Group point out that radicular pain, which occurs when nerve roots are involved, is commonly called sciatica. However, the term sciatica is often overused by doctors and patients [2].

LBP is a common condition affecting primarily populations in developed countries, causing serious problems in public health systems around the world. Data on the prevalence of LBP are divergent and range from 2% to 25% of the general population [1,3]. Acute symptoms of LBP usually appear between the ages of 20 and 40, and in 40–90% of patients, the symptoms disappear within six weeks of their onset [4]. However, the recurrence rate of LBP is high; according to Deyo and Phillips [5], approximately 20% of patients are at risk of developing a chronic syndrome and experiencing complex psychosocial and occupational problems. LBP lasting longer than 12 weeks is considered a chronic disease and is associated with the risk of sleep problems, depression and/or anxiety, and often disability. LBP is the leading cause of Years Lived with Disability (YLD) and a major challenge to primary health care worldwide [6]. On 19 June 2023, World Health Organization (WHO) posted a fact sheet on LBP in its newsroom, referring to data from an article published in Lancet Rheumatology [7]. The article, prepared by the GBD 2021 Low Back Pain Collaborators and based on a systematic analysis of the Global Burden of Disease Study 2021, discusses the global, regional, and country-level (for 204 countries) burden of LBP from 1990 to 2020, associated risk factors, and prognosis. In 2020, an estimated 619 million people suffered from LBP worldwide, and projections indicate that this number is likely to reach 843 million cases by 2050, mainly due to population growth and aging. Because LBP is the most common cause of disability worldwide, there is no larger group of patients requiring rehabilitation for recovery. The prevalence of LBP increases with age, peaking in individuals aged 50–55, and continues to rise up to the age of 80. The most common form is non-specific, idiopathic LBP—in which the significant cause of pain cannot be determined, accounting for approximately 90% of cases. This type of LBP may impair the patient’s motor functions, reduce quality of life and well-being, and consequently limit their ability to work and participate in family and social life. LBP remains a serious challenge for public health systems and a contributing factor to declining work productivity [7].

Various systems developed by neurologists, neuroradiologists, neurosurgeons, orthopedists, rheumatologists, physiotherapists, psychologists, and biomechanists, among others, are used to classify LBP. These systems are supported by statistical data and/or expert assessments and allow understanding of the mechanisms, symptoms, and psycho-logical, social, and occupational effects of LBP. Some systems, such as the Coste classification, also distinguish psychogenic low back pain [8,9]. Classification by Klippel et al. distinguishes five types of LBP: 1. Mechanical (non-specific) pain, which accounts for over 90% of all cases; 2. Inflammatory pain, which accounts for about 1% of cases; 3. Neurogenic pain; 4. Generalized, hemi- or very severe pain; 5. Psychogenic pain [10].

An important development in understanding the pathological mechanisms of pain and its transformation into a chronic form has been the advancement of knowledge about central sensitization (CS) to pain. Among the various definitions of CS, three have aroused the greatest interest: Woolf’s (“…enhancement of neural signaling in the CNS that causes hypersensitivity to pain…”), Loeser and Treede’s (“Excessive reactivity of nociceptive neurons in the central nervous system to normal or subthreshold afferent stimulus”), and Nijs et al.’s (…“excessive response of central neurons to stimulation from unimodal and polymodal receptors…”) [11,12,13]. Peripheral sensitization is associated with hyperexcitability localized in the lesion area, whereas CS occurs outside the lesion area. One of the most important symptoms of CS is allodynia, which describes a situation when a patient has unpleasant experiences (e.g., pain, a burning sensation) from stimuli that do not cause pain in healthy people [14].

Over the last twenty years, several reports on CS have been published—most of them concern LBP, pain in the musculoskeletal system, restless legs syndrome, irritable bowel syndrome, vulvodynia, headaches, and postoperative pain. CS is hypothesized to be one of the mechanisms underlying chronic pain [15]. Patients with chronic severe pain often experience accompanying symptoms such as fatigue, depression, anxiety, sleep disorders, appetite disorders, flatulence, dyspepsia, and dizziness—these are part of the picture of CS. As research progressed, it became clear that the causes of LBP involve both organic and mental aspects.

## 2. Assessment of Central Sensitization

Central sensitization (CS) is manifested by a decrease in the threshold of skin sensitivity to pain, hyperalgesia (increased and prolonged response to painful stimuli), and a secondary abnormal reaction that transfers sensitivity beyond the area of injury to un-damaged tissue. To diagnose CS pain, three main criteria must be met: (1) pain that is not proportional to the nature of the pathology and extends beyond the injury; (2) pain distribution that is widespread, with the presence of allodynia and hyperalgesia; and (3) abnormal sensitivity that is not related to the musculoskeletal system. People suffering from CS pain exhibit hypersensitivity to bright light, touch, loud sounds, pesticides, skin pressure, some medications, and extreme temperatures. In some individuals, these symptoms may also be associated with fatigue, sleep disturbances, non-restorative sleep, difficulty concentrating, a feeling of swelling in the limbs, tingling, and numbness [11,12,13].

These above-mentioned features are important in differentiating pain caused by CS from neuropathic pain, which is manifested by more or less specific symptoms and signs and is caused by several different diseases and lesions. Neuropathic pain may be caused by various disorders of both the peripheral and central nervous systems. The most common and well-known peripheral neuropathic pain syndromes include painful diabetic neuropathy, trigeminal and postherpetic neuralgia, persistent postoperative and post-traumatic pain, complex regional pain syndrome, neuropathic pain associated with cancer, neuropathic pain associated with HIV infection, and pain after amputation. Less common central pain includes central pain after a stroke, central pain following spinal cord injury, central pain in Parkinson’s disease or other neurodegenerative diseases, pain in syringomyelia, and multiple sclerosis [16,17].

In contrast to neuropathic pain, also called non-receptor pain, there is receptor pain (nociceptive), which can occur as both chronic (pathological) and acute (physiological) [18]. It is caused by stimuli that damage tissues, which in turn activate appropriate receptors—nociceptors (from Latin “to harm or hurt”). This pain is transmitted through the normal, undamaged nervous system to higher centers. In clinical practice, it is the most common type of ailment in humans and accounts for over 90% of all pain experiences [19]. Central sensitization only occurs in cases of nociceptive pain.

Three questionnaires are most commonly used to assess CS: the Central Sensitization Inventory (CSI), the Allodynia Symptom Checklist (ASC), and the self-rated Pain Sensitivity Questionnaire (PSQ) [18]. The most frequently used is the CSI (validated in twenty-seven languages), followed by the ASC (validated in eight languages) and the PSQ (validated in five languages). The CSI consists of two parts: Part A contains questions about symptoms related to CS, to which the respondent selects one of five answers, specifying 25 ailments. In part B, the respondent answers whether they have been diagnosed with one of ten syndromes: restless legs syndrome, chronic fatigue syndrome, fibromyalgia, temporomandibular joint disease, migraine or tension headaches, irritable bowel syndrome, hypersensitivity or allergy to chemicals, neck injury (including whiplash), anxiety disorders, or depression. The total result obtained in part A gives the sensitization index, which indicates the intensity of pain: subclinical (from 0 to 29 points), mild (from 30 to 39), medium (from 40 to 49), severe (from 50 to 59), and extreme (from 60 to 100) [20,21].

The Allodynia Symptom Checklist (ASC) is composed of 12 questions about activities that may cause possible allodynia. These activities include combing own hair, brushing own hair back, shaving own face, putting on glasses, putting in contact lenses, putting on earrings, fastening a necklace, putting on tight clothing, showering, placing face or head on a pillow, and exposure to heat and cold. The respondent can choose one of five answers specifying the frequency of allodynia. From the summary of results, the overall severity of allodynia is classified as follows: 0–2 points: none, 3–5: slight, 6–8: moderate, and 9 or more: severe [22]. The Self-rated Pain Sensitivity Questionnaire (PSQ) was developed as an alternative to experimental pain tests. The PSQ consists of 15 questions covering self-assessment of pain intensity after various stimuli (heat, cold, pressure, sharp pinprick) in the head, upper limbs, and lower limbs. Completing the questionnaire takes 5–10 min [23].

## 3. Central Sensitization in Low Back Pain

While the definitions of CS were clarified in 2008, 2010, and 2011, reports on central pain processing have appeared since the 1990s. In 1994, Arendt-Nielsen et al. [24] as a result of an experiment conducted on eight volunteers, described one of the mechanisms of central pain processing “wind-up” which results from repeated electrical stimulation with a frequency increasing up to supra-threshold values, causing simultaneous summation felt as increased pain. This phenomenon was repeatedly described in the following years as an increase in the intensity of pain when a given stimulus is delivered in a dose many times exceeding the critical values. In 2000, Herrero et al. [25] attempted to explain this phenomenon as a system of amplification in the spinal cord of the nociceptive message that comes from peripheral nociceptors connected to C afferent fibers. This mechanism is not fully understood and is associated with the defective functioning of spinal inhibitory interneurons (GABAergic, opiate-dependent, and serotonin-dependent). Incorrect inhibitory activity of these interneurons (in any configuration) causes excessive activity of the Wide-Dynamic-Range (WDR) neuron, where the incoming information is modulated and sent to the brain via the spinothalamic pathway. Pain is perceived in the brain, and a response to the pain stimulus is triggered in the form of a vegetative, cognitive, and affective reaction. Activation of WDR neurons stimulates not only brain centers but also, through axonal collateral connections, activates segmental centers, which in turn stimulate sympathetic centers (lateral horns of the spinal cord) and α-type motor centers. Sympathetic stimulation causes vasodilation in the dysfunctional site, which disrupts lymph drainage and contributes to the activation of trigger points in the muscles located in this area. Stimulation of motor centers, in turn, leads to muscle deactivation. Prolonged muscle deactivation promotes their trophic disorders due to ischemia and, as a result, causes pain [26]. This mechanism forms a “vicious circle” that promotes the sensitization of appropriate brain centers to stimuli coming from a specific area of the body. If we recognize that the joy of life, satisfaction, and even therapeutic touch stimulate serotonin-dependent inhibitory interneurons, which in turn have an inhibitory effect on WDR neurons, it is easy to imagine the effect that negative emotional states accompanying LBP may have on the tuning of this interaction.

In 2004, an international team of researchers led by Giesecke et al. [27] conducted a pilot study on 11 patients with idiopathic LBP persisting for at least 12 months, whose pain could not be explained by changes in MRI or X-ray. The intensity of pain sensation was assessed using a series of pressures with increasing strength, ranging from 0.5 kg/cm^2^ to tolerance, or a maximum of 10 kg/cm^2^, along with fMRI after applying a 2 kg stimulus to the same place, which caused the subjective feeling of pain to be slightly intense. The study found hyperalgesia in both the LBP group and the 16-person fibromyalgia group. The pressure required to produce mild pain intensity was noticeably greater in the control group of 11 healthy volunteers than in the LBP or fibromyalgia patients (*p* = 0.006). Five common areas of neural activation emerged on fMRI in pain-related cortical areas for both the LBP and fibromyalgia groups in area S2 of the contralateral primary and secondary somatosensory cortex, inferior parietal lobe, the cerebellum, and ipsilateral S2. This stimulus resulted in only one activation in the control group, in area S2 of the contralateral somatosensory cortex. At the same levels of pressure, the LBP or fibromyalgia patients experienced significantly more pain and more diffuse patterns of neural activation in pain-related cortical areas. The authors concluded that these results are consistent with the observation of increased central pain processing in patients with idiopathic low back pain [27].

The first comprehensive study on CS in LBP, which discussed 128 reports, was published in 2013. Roussel et al. [28] were inspired by conflicting results from studies assessing the response to various stimuli in patients with CLBP. Some of these studies showed an exaggerated pain response to sensory stimulation of sites outside the painful area, while others found no differences between patients and healthy subjects.

The prevalence of CS in people with LBP symptoms is estimated at various levels. CSI scores ≥ 40 in the LBP population were estimated to range from 18.3% to 78.2% [29,30]. In a study of 500 people with chronic musculoskeletal pain, Yücel and Saran-Toprak [31] found among 165 patients with chronic CLBP, CS was found in 72 (43.6%). Among the 165 patients with CLBP, subclinical or mild severity levels of CSI scores predominated (56.4%), while severe and extreme CS symptoms were recorded in 45 people (27.3%) [32].

Studies investigating the integrity of endogenous pain inhibitory systems have shown an unchanged direction of transmission of this descending inhibitory system. On the other hand, studies on the effects of experimentally induced pain on brain structures and function have allowed preliminary conclusions to be drawn, indicating changes in central nociceptive processing in patients with chronic LBP. Certain psychosocial features, such as inappropriate pain perception, exaggerated and excessive pain sensation, or depression, may contribute to the development of central sensitization mechanisms. Some researchers have speculated that nociception is associated with the reorganization of the cerebral cortex and subcortical territories and may influence the course of chronic LBP. This speculation is supported by structural magnetic resonance imaging (MRI) studies, which have shown reduced gray matter density in the bilateral dorsolateral prefrontal cortex (PFC) and right thalamus in individuals with CLBP [31]. Functional abnormalities in these individuals have been observed in functional MRI (fMRI) studies in specific brain regions, including the thalamus, primary and secondary somatosensory areas, anterior cingulate cortex, insular cortex, posterior cingulate cortex, and cerebellum [33,34]. Fan et al. [35] compared functional changes in the brain using fMRI in individuals with CLBP to those in healthy controls. They also assessed the relationship between functional abnormalities in the brain and pain-related behavior. For this purpose, 56 patients with CLBP and 56 healthy controls were included in the study. Pain intensity in patients with CLBP was assessed using the numerical rating scale (NRS), and pain-related behaviors were assessed using the Pain Catastrophizing Scale (PCS) and the Pain Vigilance and Awareness Questionnaire (PVAQ). Bipolar electrical stimulation was used to determine the pain threshold in both the CLBP and healthy control groups. These studies found that CLBP patients exhibited higher sensitivity, attention, and catastrophizing tendencies compared to healthy controls. Significantly higher voxel-wise amplitude of low-frequency fluctuation was recorded in different brain regions within the so-called pain matrix and default mode network in CLBP patients. These changes correlated positively with pain intensity (R = 0.51, *p* < 0.001) and negatively with pain sensitivity (R = −0.43, *p*< 0.001). These findings provide direct evidence that individuals with CLBP exhibit pain hypersensitivity associated with functional abnormalities in the brain. The results of these studies suggest that imaging markers could be helpful in detecting individuals with central pain sensitization [34]. It is hoped that future prospective studies using fMRI will be able to clarify whether the described changes are reversible and whether they are associated with functional improvement [28,35].

Corrêa et al. [36] investigated changes in local and segmental hypersensitivity and endogenous pain inhibition in 30 people with chronic non-specific LBP. Pressure Pain Thresholds (PPT) were measured using a pressure of 50 kPa on an area of 1 cm^2^ in the paralumbar region and on the tibialis anterior muscle, and the Conditioned Pain Modulation (CPM) activation index was measured using a cold test by immersing the patients’ foot in a bucket of ice water. Compared to a control group of healthy volunteers, people with LBP had significantly lower PPT in both the lumbar region and 30 s after foot immersion in ice water, particularly in women [36].

In 2016, Bid et al. [37] published the results of a narrative review of 16 pieces of literature on CS and altered central pain processing in patients with CLBP, dating from 1990–2010. The results of studies assessing reactivity to various types of stimuli in patients with LBP were contradictory. Some studies showed an increased risk of pain response after sensory stimulation of a body part outside the pain region, whereas others found no differences between patients and healthy controls. Studies assessing the integrity of endogenous pain systems describe unchanged activity in the descending pathway of the pain inhibitory system. On the other hand, studies on brain structure and function in relation to experimentally induced pain have provided preliminary evidence for altered central pain processing in patients with LBP. In addition, inappropriate beliefs about pain, depression, and/or pain catastrophizing may lead to the development of CS [37].

Gräper et al. [38] developed a prognostic model of the risk of developing central sensitization symptoms based on the assessment of the sensory profile. For this purpose, 114 patients with acute LBP symptoms were studied over a 12-week period. Four sensory profiles and their thresholds were distinguished by assessing taste, smell, sight, hearing, touch, activity stimulation, and the individual’s adaptive reactions to excessive or insufficient stimulation by sensory information. Based on this assessment, the following types were distinguished: Low Registration (high sensory threshold, passive reaction), Sensation Seeking (high sensory threshold, active reaction), Sensory Sensitive (low sensory threshold, passive reaction), and the Sensation Avoiding type (low sensory threshold, active avoidance of stimulation) [39]. In addition to the sensory profiles, the independent variables included: state and trait anxiety, age, duration, pain intensity, depressive symptoms, and pain catastrophizing. These studies demonstrated a model dominated by two variables: sensory sensitivity (unstandardized B value = 0.42; *p* = 0.01) and trait anxiety (unstandardized B value = 0.53; *p* ≤ 0.001). In the case of continuous data, the multivariate linear model yielded R^2^ = 0.38, indicating that 38% of the variance in central sensitization symptoms were explained by the predictors of sensory sensitivity and trait anxiety. It was also indirectly shown that 62% of the variance in the trait of central sensitization remains unknown. Nevertheless, the authors concluded that the model, based on a sensory profile associated with sensory sensitivity (expressed as a combination of a low sensory threshold and a passive response to excessive sensory stimulation), combined with an elevated level of anxiety, was the most predictive of the development of central sensitization symptoms after 12 weeks of LBP symptoms. On this basis, it was suggested that the described model may be a useful tool in clinical settings to help predict chronic pain conditions [38].

In 2018, Huysmans et al. [40] published the results of a cross-sectional study assessing the association between CS symptoms and important cognitive-behavioral and psychosocial factors in a group of 38 patients suffering from nonspecific low back pain for at least three months. They investigated the following assessments: 1MSCT (1-min stair-climbing test); Brief IPQ (Brief Illness Perception Questionnaire); CSI (Central Sensitization Inventory); PCS (Pain Catastrophizing Scale); QBPDS (Quebec Back Pain Disability Scale); TSK (Tampa Scale For Kinesiophobia); VAS 7D (Visual Analog Scale past 7 days); and VAS NOW (Visual Analog Scale at this moment). The authors observed a significant relationship between CS symptoms and all other outcomes. Patients exhibiting more characteristic symptoms of CS syndrome performed significantly worse on most tests compared to the subgroup of patients with fewer symptoms of CS syndrome [40].

Aoyagi et al. [41] studied a total of 46 patients aged 21 to 70 years, an average of 43 (70% female), suffering from LBP for at least three months. Twenty-two (48%) participants were identified as meeting the criteria for a diagnosis of fibromyalgia (FM) according to the 2011 Fibromyalgia (FM) Survey, which consists of two parts: the Widespread Pain Index (WPI) and Symptom Severity (SS). WPI assesses whether the subject feels pain or tenderness in 19 specific places on the body (score 0–19), while SS assesses the severity of symptoms on a scale of 0–12. According to these criteria, a patient can be diagnosed with FM if the test results are ≥7 on the WPI scale and ≥5 on the SS scale, or if the WPI score is between 3 and 6 and the SS score is ≥9. Since 2015, the FM 2011 Test, which is a form of self-assessment, has become the most frequently used target for the diagnosis of CS28 in scientific research. FM patients showed lower levels of PPT (Pressure Pain Thresholds) in the thumb and lumbar spine, lower values of CPM (Conditioned Pain Modulation) in the thumb, and more marked symptoms of inappropriate pain perception, excessive pain perception, and higher levels of anxiety and depression than LBP patients without FM. Pathomechanism was found to be significantly correlated with PPT and CPM, and with psychosocial symptoms. According to these results, the authors suggest the existence of a subgroup of LBP patients who exhibit symptoms and signs of CS. The relationship between subjective and objective values of CS indicates that the FM 2011 test can be used to identify CS in LBP patients in clinical practice [41,42].

Little work has focused on attempts to explain the pathomechanism of CS formation in LBP. Li et al. [43] published a comprehensive narrative review article on the peripheral and central pathological mechanisms of chronic LBP (lasting >3 months). Considering the heterogeneity of LBP pathology, the mechanisms of its development are complex and difficult to understand. The authors stated that the central mechanism of chronic LBP is the alteration of brain sensory processing and abnormal functioning of the descending pain modulation system, which causes pain amplification in the central nervous system (CNS). Abnormalities in brain biochemical metabolism, activation of glial cells, and subsequent inflammation also play important roles. The authors believe that inflammation plays an key role in both peripheral and central sensitization of LBP. Future studies on the individual components of LBP pathogenesis should distinguish specific forms of the disease and identify its origin, which will allow the development of effective therapy against chronic LBP [43].

Since no direct pathoanatomical cause of pain could be detected in most patients with CLBP symptoms, Sirucek et al. [44] attempted to assess signs of peripheral, spinal, and supraspinal sensitization in 59 patients with CLBP. To do this, they applied a quantitative sensory test to the most painful area, the adjacent non-painful area, and to 35 healthy controls on the side of the nondominant hand. Normal sensory integrity was assessed in all participants through bedside sensory testing of vibration, temperature, pinprick, and light touch. Control participants were assessed at the same test sites as the patient with CLBP to whom they were matched. The reduced sensitization assessment protocol included cold pain thresholds, heat pain thresholds, mechanical pain thresholds, mechanical pain sensitivity, dynamic mechanical allodynia, wind-up ratio, and pressure pain thresholds. Patients with CLBP showed cold and vibration hypoesthesia in the most painful area (both *p* < 0.001), mechanical hyperalgesia (*p* < 0.001), and a higher incidence of dynamic mechanical allodynia (*p* = 0.044) in the area adjacent to the most painful area. This distribution of results was most influenced by the subgroup of patients with CLBP with high pain intensity. No sensory changes were observed in the control group. The authors concluded that mechanical hyperalgesia and dynamic mechanical allodynia in the back area adjacent to the painful area may reflect secondary hyperalgesia originating from spinal cord sensitization in patients with CLBP [44]. These results indirectly correlated with the results of a randomized, double-blind, controlled experiment conducted in young, healthy individuals [45]. In one group, acute nociceptive pain of the lower back was induced by the injection of physiological saline. In the first control group, a sham injection of physiological saline (without piercing the skin), potentially inducing nocebo pain, was performed, while in the second control group, no intervention was performed. Touch acuity was measured using the two-point discrimination threshold (TPD), point-to-point test (PTP), and two-point estimation task (PTE) before, during, and after pain relief. TPD was found to be worse during pain induction in the experimental group compared to the control group (*p* < 0.001) and increased on average by 7.28 mm during pain perception. Maximum reported pain was a significant predictor (β = 0.55; *p* = 0.01) and accounted for 26% of the variance for TPD (*p* < 0.05). The results of the remaining tests (PTP and PTE) showed a similar trend of changes, but were not statistically significant. In conclusion, the authors of this medical experiment stated that acute nociceptive pain linearly worsens the sharpness of touch [45].

Echeita et al. [46] demonstrated the importance of recognizing CS in vocational rehabilitation. The article begins by quoting Bevan [47], who stated that in Europe, 50% of absenteeism and 60% of permanent work disability are due to musculoskeletal disorders, including LBP [47]. They conducted a cross-sectional, multicenter observational study involving 56 patients, with an average age of 42.5 years, including 59% women. Waddell’s signs, which are prognostic factors after spine surgery, were used. These include eight tests, described in 1980 as Non-organic Signs (NOS), which indicate the psychogenic origin of symptoms, aggravation, or simulation [48]. The ability to lift weights was assessed using the WorkWell protocol, described in 2004 by Gross et al. in which the subject is asked to remove a box with increasing load from a height of 75 cm [49]. The correlations between maximum load capacity and CSI and NOS were r= −0.53 and r= −0.50, respectively. CS and NOS, as well as age and gender, contributed significantly to the final regression model, which explained 57.6% of the variance. After adjusting for confounding factors, CS and NOS were found to be negatively associated with lifting capacity in patients with LBP. The results of this study allow for the creation of optimized vocational rehabilitation programs and faster return of patients to work [49].

Akeda et al. [50] from Japan conducted a study on 272 residents living in a mountain village. The average age of the study participants was 72.1 years, and 28.3% of them complained of chronic LBP. The pain numerical score (NRS), Oswestry Disability Index (ODI) and CSI scores were significantly higher in the group complaining of LBP than in the control group (without LBP). An essential relationship was found between the CSI scores and the NRS, ODI, and quality of life assessed by EQ5D and EQ-VAS. It was shown that disability, pain, quality of life, and age were the factors significantly associated with CSI. The results of this study led the authors to conclude that CS is associated with LBP [50].

There are a number of papers on pharmacological and non-pharmacological treatments for CS. In the pharmacotherapy of patients with a dominant CS component in LBP, drugs that affect central mechanisms—such as antidepressants from the SSRI group and tricyclic antidepressants—may be effective, while classic anti-inflammatory drugs will not be effective. In physiotherapy, it is important to remember that interventions aimed at relieving pain at a local level—within the painful anatomical area—are usually of limited benefit in these cases. Because patients experience a lot and focus on pain, treatment should begin with education, followed by a phase of stress reduction interventions. Cognitive-behavioral therapy will play a fundamental role at this time, with the goal of helping the patient develop strategies for coping with pain and related emotions, such as anger or a sense of injustice. Nijs et al. [51] presented an expert opinion on pharmacotherapy in CS in 2019. The available evidence is insufficient to recommend the standard use of centrally acting drugs, such as tricyclics, serotonin-norepinephrine reuptake inhibitors, and α2δ ligands, in patients with chronic pain. This is not surprising, as CS is associated with complex psycho-neuro-immunological interactions, and each of the tested drugs affects one or two of these mechanisms from a purely biomedical point of view. Under-standing central sensitization means clarifying the concept of CS, focusing on the long-term rather than short-term effects of treatment, and addressing perpetuating factors such as stress, sleep problems, physical inactivity, inadequate cognitive functions, and/or dietary factors [51].

Pardos-Gascón et al. [52] conducted a systematic review of mindfulness-based interventions (MBI) and cognitive behavioral therapy (CBT) for chronic pain and its consequences. Eighteen studies met the inclusion criteria; four studies were found for chronic LBP. In terms of physical function and pain intensity, mindfulness-based stress reduction (MBSR) was more effective than standard care, but not better than CBT in LBP [52]. Adams and Turk [53] emphasized that CS constitutes a heterogeneous group of disorders characterized by chronic pain. They noted that biological, psychological, and social factors operate independently and together influence a person’s experience. The authors noted a disparity between psychosocial factors as causes of symptoms and modifiers and perpetuators of symptoms. They cautioned against viewing all patients with the same diagnosis as a homogeneous group. The experience of pain is largely private, highly personal, and subjective—it is multifactorial, encompassing physiological, behavioral, cognitive, affective, and social features [52]. Bao et al. [54] recently presented a review of neuroimaging mechanisms in cognitive behavioral therapy (CBT) for pain management. Current neuroimaging studies of CBT have used structural and fMRI to analyze changes in brain gray matter volume, as well as activation and deactivation of brain regions and intrinsic connectivity between them. The results showed that after CBT, the brain exhibited stronger top-down pain control, cognitive reappraisal, and altered perception of stimulus signals. The authors concluded that brain areas such as the dorsal lateral prefrontal cortex, orbitofrontal cortex, ventral lateral prefrontal cortex, posterior cingulate cortex, and amygdala may be crucial for the response during cognitive-behavioral interventions for pain treatment [53].

## 4. Summary

Schuttert et al. [55] conducted a systematic review of 34 studies on the definition, assessment, and prevalence of CS in patients with chronic LBP. Since central pain sensitization cannot be directly assessed, they proposed the term Human Assumed Central Sensitization (HACS). Forty different references were used to define HACS. This review identified twenty quantitative methods of assessing HACS, including four questionnaires and sixteen quantitative sensory tests. The prevalence of HACS in patients with chronic low back pain was estimated in three studies. It was emphasized that the literature on HACS in patients with CLBP contains many definitions, assessment methods, and incidence estimates. While most HACS assessment methods are not validated, they have been tested for reliability and repeatability. Given the lack of a gold standard for HACS assessment, the authors propose the creation of a unified classification system for the clinical and research assessment of HACS in patients with chronic LBP [55].

Low back pain (LBP) is a major challenge and a significant burden for healthcare systems. Thanks to the discovery of the phenomenon of central sensitization (CS) to pain, we have gained a better understanding of the pathophysiology of pain and the transformation of LBP into a chronic form. However, there is still no standard system for recognizing and assessing CS. The concept of creating a unified classification system for the clinical and research assessment of HACS in patients with chronic LBP should be considered. Answering the question posed in the title: Central Sensitization can be seen as an excessive reactivity of nociceptive neurons in the central nervous system to normal or sub-threshold afferent chronic stimuli in people with certain mental predispositions. Further randomized, well-designed studies with large sample sizes are necessary to better elucidate the mechanisms of CS.
